# Supplementary material for: A Dual-Signaling Electrochemical Aptasensor Based on an In-Plane Gold Nanoparticles–Black Phosphorus Heterostructure for the Sensitive Detection of Patulin
Source: Foods. 2023 Feb 16;12(4):846. doi: 10.3390/foods12040846 (PMC9957366; doi:10.3390/foods12040846)
Supplement: Supplementary file 1 [file foods-12-00846-s001.zip › foods-2191417-supplementary.pdf]

Supporting Information

A Dual-Signaling Electrochemical Aptasensor Based on an In-Plane Gold Nanoparticles–Black Phosphorus Heterostructure for the Sensitive Detection of Patulin

Jinqiong Xu [1], Jianbo Liu [1], Wuwu Li [1], Yongsheng Wei [1], Qinglin Sheng [2,3,*] and Yonghui Shang [1,*]

[1] College of Chemistry and Chemical Engineering, Xianyang Normal University, Xianyang 712000, China
[2] College of Food Science and Technology, Northwest University, Xi'an 710069, China
[3] Laboratory of Nutritional and Healthy Food-Individuation Manufacturing Engineering/Research Center of Food Safety Risk Assessment and Control, Xi'an 710069, China
* Correspondence: qlsheng@nwu.edu.cn (Q.S.); shangyh@xync.edu.cn (Y.S.)

Supplementary details

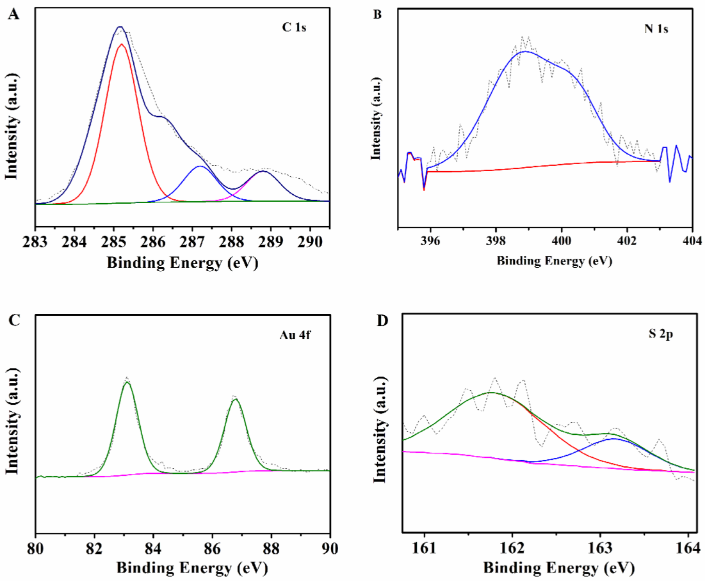

Figure S1. High-resolution XPS spectra for (A) C 1 s, (B) N 1 s, (C) Au 4f, and (D) S 2p regions collected from aptamer compound AuNPs-BPNS.

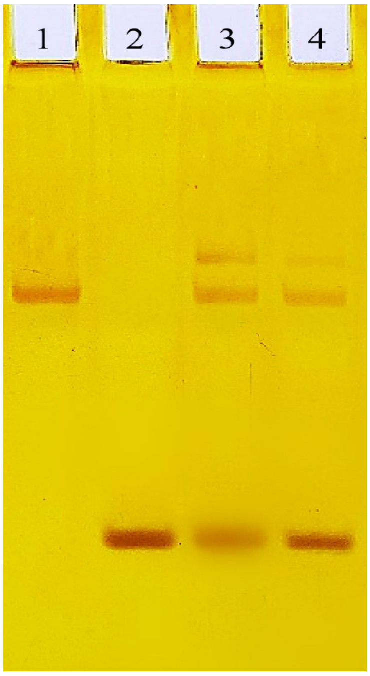

Figure S2. PAGE images of different DNAs. Lane 1, aptamer; Lane 2, cDNA; Lane 3, hybridization of aptamer and cDNA; Lane 4, hybridization of aptamer and cDNA treated with PAT.

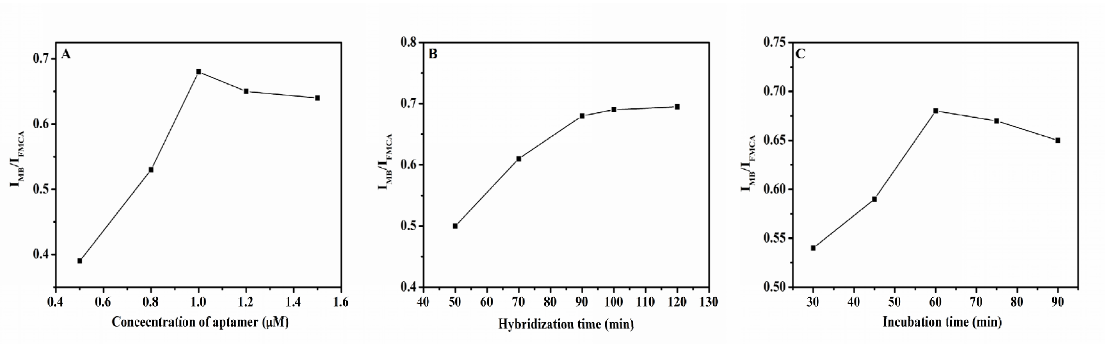

Figure S3. Optimization of the concentration of aptamer (A), the hybridization time of aptamer and cDNA (B) and the incubation time of PAT (C) for the PAT (154 ng/mL) detection.
